# Trends in skin cancer incidence by socioeconomic position in Scotland, 1978–2004

**DOI:** 10.1038/sj.bjc.6605678

**Published:** 2010-05-04

**Authors:** V R Doherty, D H Brewster, S Jensen, D Gorman

**Affiliations:** 1 Department of Dermatology, Royal Infirmary of Edinburgh, Lauriston Place, Edinburgh EH3 9HA, UK; 2 Scottish Cancer Registry, Information Services Division, NHS National Services Scotland, Gyle Square, 1 South Gyle Crescent, Edinburgh EH12 9EB, UK; 3 Cancer Surveillance Team, Information Services Division, NHS National Services Scotland, Gyle Square, 1 South Gyle Crescent, Edinburgh EH12 9EB, UK; 4 Department of Public Health, NHS Lothian, 148 The Pleasance, Edinburgh EH8 9RS, UK

**Keywords:** socioeconomic status, skin cancer, trends

## Abstract

**Background:**

Non-melanoma skin cancer has been little studied in relation to deprivation.

**Methods:**

Incident cases diagnosed in 1978–2004 were extracted from the Scottish Cancer Register and assigned to quintiles of Carstairs deprivation scores. Age-standardised incidence rates (ASRs) (European standard population) were calculated by deprivation quintile, sex, period of diagnosis, for the three main types of skin cancer.

**Results and conclusion:**

As age-standardised incidence of each skin cancer increased significantly over time across all deprivation categories, rates were consistently highest in the least deprived quintile.

In the past three decades, reported incidence of cutaneous melanoma (CM) has risen worldwide, in both high- and low-incidence areas, such as Australia (Curado *et al*, 2007) and Holland and Scotland (deVries and Coebergh, 2004; Mackie *et al*, 2007), respectively. Although reporting of non-melanoma skin cancers (NMSCs) is recognised to be less complete than that for CM, studies from Northern Ireland and Scotland have shown a parallel rise also in these (Brewster *et al*, 2007; Hoey *et al*, 2007).

Melanoma, along with breast cancers, is recognised to be one of the few malignancies that are more common among the more affluent (Information Services Division website, 2009). However, as in other malignancies, CM survival is poorer in more deprived groups, mirroring the survival ‘deprivation gap’ in breast cancer (Shack *et al*, 2007). This deprivation effect was reported for CM in west Scotland, where relatively poorer prognosis was noted for patients from deprived backgrounds (MacKie and Hole, 1996). An examination of CM in 1996–2004 by postcode by the Thames Cancer Registry found that incidence was highest in both sexes in the most affluent groups (Grunewald *et al*, 2007), perhaps reflecting their easier access to ‘sunshine holidays’. In the past two decades, there has been a large increase in budget airline travel offerring either increased availability of sunshine holidays for more deprived groups, with narrowing of the deprivation gap, or a further increase in such holidays for the less deprived, resulting in a widened gap. Alternatively, all groups may now take more sunny holidays, with no change in the gap. At present, there is no accessible information on who uses budget air travel.

The aetiology of NMSC, that is, basal cell (BCC) and squamous cell (SCC) cancers, has been considered to differ from CM, being more frequently associated with chronic sun exposure and outdoor occupations, whereas CM has been related to intermittent, recreational sun exposure. More recently, a dual aetiological hypothesis for CM proposes that two patterns of melanoma may exist, one reflecting chronic sun exposure similar to NMSCs and another reflecting intermittent, intense exposure (Whiteman *et al*, 2003). Thus one might expect similarities in the patient demographics between at least some melanomas and NMSCs.

Little work has been carried out on deprivation in NMSC. In Northern Ireland, BCC and CM were more frequent among less deprived individuals (Hoey *et al*, 2007). We examined our data for any changing pattern of deprivation in the three main types of skin cancer in Scotland over a 27-year period.

## Materials and methods

Anonymised records of incident cases of BCC, invasive SCC of the skin and invasive CM, spanning the period of diagnosis 1978–2004, were extracted from the Scottish Cancer Registry database. On the basis of postcode sectors of residence, individuals were assigned to quintiles of Carstairs deprivation scores by applying 1981, 1991 and 2001 census-derived Carstairs scores to the periods of diagnosis 1978–1985, 1986–1995, 1996–2004, respectively. The Carstairs deprivation index is based on small area of residence, and is derived from four variables collected at each decennial census: social class, unemployment, overcrowding and car ownership (Morris and Carstairs, 1991). Previous work has shown that, when studying trends in socioeconomic inequalities since the mid-1970s, the use of the 1981, 1991 and 2001 Carstairs scores applied to successive time periods provides similar results to the use of the 1991 index on its own (Conway *et al*, 2007). Mid-year population estimates were obtained from the General Register Office for Scotland (Registrar General for Scotland, 1979–2005). Age-standardised incidence rates (ASRs), standardised to the European standard population, were calculated by deprivation quintile, sex, 3-year period of diagnosis and type of skin cancer. Poisson regression modelling was used to assess the magnitude and statistical significance of differences in incidence rates. Summary data showing numbers of cases and ASRs in earliest and most recent 3-year periods were prepared.

## Results

For all three types of skin cancer there was a statistically significant increase in age-standardised rates over time. Tables 1,2,3 give condensed results comparing numbers of cases and ASRs for the earliest and most recent 3-year periods. Fuller Tables 4,5,6 showing ASRs for all time periods are available online. For the total period of observation, the incidence of all three major skin cancers was higher in the least deprived than in the most deprived quintile. These trends were all significant except in females with invasive SCC (*P* for trend values: melanoma males 0.0003, females 0.0001; SCC males 0.0046, females 0.7071; BCC males 0.001, females 0.022). The ratio of incidence rate in quintile one to that in quintile five (deprivation ratio) was calculated as a measure of socioeconomic difference; thee deprivation ratio for CM was greater than for NMSC. There were no statistically significant interactions between deprivation and period of diagnosis for any of the three cancer types (all *P*>0.05), indicating no significant widening or narrowing of inequalities in incidence over time.

## Discussion

This Scottish data not unexpectedly confirms the ongoing rise of all three types of skin cancer over a 27-year period. Case ascertainment may have increased over time, especially with increased availability of electronic pathology records. For all types, UV exposure is considered to be the main aetiological factor (Armstrong and Kricker, 2001). Notably, despite arguments about differing aetiologies for CM and NMSC, the distribution of cases across the deprivation categories is similar for all three cancer types, suggesting some degree of shared aetiology.

For CM alone, there has been no significant change in the deprivation category 1 : 5 ratio over the study period. It has been suggested that the variation across deprivation categories for CM reflected intermittent high sun exposure such as during sun-seeking holidays. The recent increased availability of budget airline travel could have been expected to extend such exposure to a wider spread of socioeconomic groups. However, we found no change in the deprivation ratio over a 27-year period suggesting that either all groups are now having more sun or that the persisting socioeconomic differences have other causes.

For NMSC, classically associated with chronic sun exposure, it is surprising that a similar gradation to CM is demonstrated across deprivation categories. It might have been expected that greater numbers of outdoor workers would be found in categories 4 and 5 giving the opposite situation to CM. For these cancers too, the deprivation ratio has not altered over time. The Northern Ireland study, which looked at workload implications of the rising incidence of skin cancer, covered a shorter period (1993–2004) (Hoey *et al*, 2007), and noted similar rising trends for all three cancer types. Deprivation was measured using a somewhat similar scoring system to our analyses. However, they found no correlation between SCC and deprivation, whereas both BCC and CM were more frequent in the less deprived as was found in Scotland.

Our results on skin cancer contrast with oral cancer in Scotland, which showed a clearly increasing deprivation category 5 : 1 ratio over a similar time period (Conway *et al*, 2007). In oral cancer, in which smoking is an accepted risk factor, the change was more marked in males and the decline of smoking in the less deprived groups was considered to have contributed.

This study demonstrates further similarities between CM and NMSC in both changing incidence and socioeconomic distribution. The lack of change in deprivation ratio over time weighs against attributing all skin cancer entirely to sun exposure, and further work is indicated.

## Figures and Tables

**Table 1:** Invasive cutaneous melanoma: age-standardised incidence rates (ASR) by sex, deprivation quintile and calendar period of diagnosis

**Cutaneous melanoma (International Classification of Disease ICD-10 C43)**			
**Sex**	**1978–80**	**2002–04**	***P*-value for trend over time**
*Males*			
Number of cases	266	1079	<0.001
ASR – least deprived	4.8	17.2	
ASR – most deprived	2.4	9.7	
Ratio quintiles 1 : 5	2.0	1.8	
			
*Females*			
Number of cases	548	1446	<0.001
ASR – least deprived	9.2	17.3	
ASR – most deprived	4.5	12.4	
Ratio quintiles 1 : 5	2.1	1.4	

Abbreviations: ASR=age-standardised incidence rates; ICD=International Classification of Diseases.

**Table 2:** Invasive squamous cell carcinoma of skin: age-standardised incidence rates by sex, deprivation quintile and calendar period of diagnosis

**Squamous cell carcinoma (ICD-10 C44, ICD-O-2 807)**			
**Sex**	**1978–80**	**2002–04**	***P*-value for trend over time**
*Males*			
Number of cases	1049	3324	<0.001
ASR – least deprived	15.4	42.2	
ASR – most deprived	14.7	31.5	
Ratio quintiles 1 : 5	1.0	1.3	
			
*Females*			
Number of cases	696	2224	<0.001
ASR – least deprived	6.7	15.5	
ASR – most deprived	6.7	12.0	
Ratio quintiles 1 : 5	1.0	1.3	

Abbreviations: ASR=age-standardised incidence rates; ICD=International Classification of Diseases.

**Table 3:** Basal cell carcinoma of skin: age-standardised incidence rates by sex, deprivation quintile and calendar period of diagnosis

**Basal cell carcinoma (ICD-10 C44, ICD-O-2 809)**			
**Sex**	**1978–80**	**2002–04**	***P*-value for trend over time**
*Males*			
Number of cases	2420	8399	<0.001
ASR – least deprived	41.6	120.4	
ASR – most deprived	29.7	88.4	
Ratio quintiles 1 : 5	1.4	1.4	
			
*Females*			
Number of cases	2579	8173	<0.001
asr – least deprived	27.1	78.9	
ASR – most deprived	25.0	63.3	
Ratio quintiles 1 : 5	1.1	1.2	

Abbreviations: ASR=age-standardised incidence rates; ICD=International Classification of Diseases.

**Table 4:** Invasive cutaneous melanoma: age-standardised incidence rates by sex, deprivation quintile and calendar period of diagnosis

**Sex**	**Deprivation quintile**	**1978–80**	**1981–83**	**1984–86**	**1987–89**	**1990–92**	**1993–95**	**1996–98**	**1999–01**	**2002–04**	***P*-value for trend over time**
Males	All	3.9	4.3	6.0	7.6	8.2	8.9	10.9	11.0	13.2	<0.001
	1	4.8	6.1	7.8	10.1	11.4	12.3	13.9	14.6	17.2	<0.001
	2	4.2	4.9	6.0	8.4	9.5	10.1	11.2	12.4	13.5	0.002
	3	4.3	3.6	6.5	7.2	6.7	8.1	9.5	11.1	13.5	0.003
	4	4.1	4.6	5.8	7.1	7.5	7.2	10.4	8.9	12.1	0.016
	5	2.4	2.3	4.1	5.3	5.9	6.6	9.4	8.1	9.7	0.001
Ratio 1 : 5		2.0	2.7	1.9	1.9	1.9	1.9	1.5	1.8	1.8	—
											
Females	All	6.4	6.8	9.2	9.8	10.4	12.3	13.0	12.6	15.0	<0.001
	1	9.2	9.1	12.6	14.1	14.0	17.3	18.9	16.9	17.3	0.016
	2	6.7	8.2	10.5	10.5	10.8	14.8	13.7	11.9	17.6	0.011
	3	5.6	6.4	9.0	9.3	11.0	10.2	13.0	12.4	14.5	0.011
	4	6.1	6.3	9.2	8.3	9.1	9.9	11.1	11.3	13.4	0.038
	5	4.5	4.0	4.8	6.9	7.1	9.1	8.3	10.3	12.4	0.006
Ratio 1 : 5		2.1	2.3	2.6	2.0	2.0	1.9	2.3	1.6	1.4	—

**Table 5:** Invasive squamous cell carcinoma of skin: age-standardised incidence rates by sex, deprivation quintile and calendar period of diagnosis

**Sex**	**Deprivation quintile**	**1978–80**	**1981–83**	**1984–86**	**1987–89**	**1990–92**	**1993–95**	**1996–98**	**1999–01**	**2002–04**	***P*-value for trend over time**
Males	All	16.1	15.8	16.6	20.4	22.8	27.0	36.1	33.9	36.9	<0.001
	1	15.4	19.5	20.5	22.8	26.1	32.2	38.8	36.7	42.2	<0.001
	2	17.7	17.5	16.6	23.0	24.6	30.4	39.1	36.2	41.2	<0.001
	3	20.0	15.6	16.8	20.0	21.5	26.2	34.4	33.4	36.4	<0.001
	4	12.6	13.0	14.2	19.1	23.1	26.5	35.3	32.7	33.4	<0.001
	5	14.7	13.1	15.1	16.9	18.4	19.6	32.8	30.8	31.5	<0.001
Ratio 1 : 5		1.0	1.5	1.4	1.4	1.4	1.6	1.2	1.2	1.3	—
											
Females	All	6.3	6.2	7.0	7.7	8.9	10.8	14.1	14.5	13.8	<0.001
	1	6.7	6.0	8.1	8.2	9.4	10.6	12.3	14.1	15.5	0.004
	2	6.2	7.0	6.8	7.3	9.5	14.2	13.8	15.5	15.0	0.001
	3	6.6	6.3	7.6	8.3	8.8	10.0	14.1	13.5	14.3	0.008
	4	5.4	6.0	6.6	7.6	8.2	10.5	14.6	14.3	12.3	0.004
	5	6.7	5.7	5.9	6.8	8.3	8.9	15.8	14.9	12.0	0.004
Ratio 1 : 5		1.0	1.1	1.4	1.2	1.1	1.2	0.8	0.9	1.3	—

**Table 6:** Basal cell carcinoma of skin: age-standardised incidence rates by sex, deprivation quintile and calendar period of diagnosis

**Sex**	**Deprivation quintile**	**1978–80**	**1981–83**	**1984–86**	**1987–89**	**1990–92**	**1993–95**	**1996–98**	**1999–01**	**2002–04**	***P*-value for trend over time**
Males	All	35.6	42.5	46.6	55.1	61.0	74.4	86.1	86.6	97.5	<0.001
	1	41.6	47.3	58.1	63.1	70.3	89.7	94.1	104.2	120.4	<0.001
	2	37.6	43.4	48.1	58.1	61.7	77.2	89.2	84.7	97.7	<0.001
	3	35.7	41.3	47.3	53.6	59.5	73.8	81.8	80.3	93.0	<0.001
	4	33.5	41.0	43.2	52.3	57.9	67.1	81.4	82.7	88.0	<0.001
	5	29.7	39.7	36.3	48.7	55.5	64.1	83.8	81.2	88.4	<0.001
Ratio 1 : 5		1.4	1.2	1.6	1.3	1.3	1.4	1.1	1.3	1.4	—
											
Females	All	25.7	29.4	35.1	40.7	46.1	54.7	65.7	65.6	67.4	<0.001
	1	27.1	32.3	39.5	45.3	54.0	58.7	69.7	74.6	78.9	<0.001
	2	26.2	29.1	36.7	41.1	46.1	54.5	65.9	65.9	70.0	<0.001
	3	25.6	29.5	36.2	40.4	44.4	56.1	60.4	64.5	65.5	<0.001
	4	24.3	28.4	34.0	36.8	44.0	53.4	60.9	59.8	59.5	<0.001
	5	25.0	27.8	29.2	40.0	42.1	50.9	71.4	63.3	63.3	<0.001
Ratio 1 : 5		1.1	1.2	1.4	1.1	1.3	1.2	1.0	1.2	1.2	—
